# Balancing exploration and exploitation in transferring research into practice: a comparison of five knowledge translation entity archetypes

**DOI:** 10.1186/1748-5908-8-104

**Published:** 2013-09-05

**Authors:** Eivor Oborn, Michael Barrett, Karl Prince, Girts Racko

**Affiliations:** 1 Warwick Business School, The University of Warwick, Coventry CV4 7AL, UK; 2 Judge Business School, University of Cambridge, Cambridge CB2 1AG, UK

**Keywords:** Knowledge translation, Exploration, Exploitation, Ambidexterity, Collaboration, Research implementation, Absorptive capacity, Innovation

## Abstract

**Background:**

Translating knowledge from research into clinical practice has emerged as a practice of increasing importance. This has led to the creation of new organizational entities designed to bridge knowledge between research and practice. Within the UK, the Collaborations for Leadership in Applied Health Research and Care (CLAHRC) have been introduced to ensure that emphasis is placed in ensuring research is more effectively translated and implemented in clinical practice. Knowledge translation (KT) can be accomplished in various ways and is affected by the structures, activities, and coordination practices of organizations. We draw on concepts in the innovation literature—namely exploration, exploitation, and ambidexterity—to examine these structures and activities as well as the ensuing tensions between research and implementation.

**Methods:**

Using a qualitative research approach, the study was based on 106 semi-structured, in-depth interviews with the directors, theme leads and managers, key professionals involved in research and implementation in nine CLAHRCs. Data was also collected from intensive focus group workshops.

**Results:**

In this article we develop five archetypes for organizing KT. The results show how the various CLAHRC entities work through partnerships to create explorative research and deliver exploitative implementation. The different archetypes highlight a range of structures that can achieve ambidextrous balance as they organize activity and coordinate practice on a continuum of exploration and exploitation.

**Conclusion:**

This work suggests that KT entities aim to reach their goals through a balance between exploration and exploitation in the support of generating new research and ensuring knowledge implementation. We highlight different organizational archetypes that support various ways to maintain ambidexterity, where both exploration and exploitation are supported in an attempt to narrow the knowledge gaps. The KT entity archetypes offer insights on strategies in structuring collaboration to facilitate an effective balance of exploration and exploitation learning in the KT process.

## Background

In recent years, the practice of translating knowledge from research into clinical practice has emerged to be of significant importance [1-5]. The rapid pace of innovation and research in the health and medical field heightens the imperative of minimizing this ‘knowledge gap’ with funding agencies allocating significant resources to close the gap. This has led to the development of new organizations that promote knowledge translation (KT) research and activity [4,6,7]. In the United Kingdom (UK), the arrival of Collaborations for Leadership in Applied Health Research and Care (CLAHRC) is one such important initiative with nine loosely coupled organizations in partnership receiving approximately £10 M new funding over a five-year duration from 2008 to 2013. Funded by the National Institute for Health Research (NIHR), the stated mission of the CLAHRCs is ‘to undertake high-quality applied health research focused on the needs of patients and to support the translation of research evidence into practice in the NHS (National Health Service)’ [8]. Each of the nine CLAHRCs is a regionally-based, NHS-led consortia pulling in external monies alongside matched funds from local partners. The exact structure of each CLAHRC emerged from the local partnership arrangements, and in particular the academic partners, that has led to the natural diversity in organizational form. There was an explicit need to equip ‘the NHS to harness better the capacity of higher education to support initiatives to enhance the effectiveness and efficiency of clinical care’ [9]. Member organizations within the partnership have unique skills, knowledge, resources, and capabilities; these are salient for conducting research, training, and changing current health service provision through implementation activities.

The majority of research into KT activity focuses on efforts by health providers to enable KT within their organizational context, or within specific clinical areas, such as oncology [10,11] or mental health delivery [12]. There are also many health service studies that describe and examine barriers and facilitators of evidence adoption across a range of policy areas [13-15]. A handful of studies have examined designated KT entities, notably the Genetics Parks established in the UK to develop the healthcare services in the area of genetics [16]. Similarly, ‘SEARCH Canada’ was established to support region-wide KT activity in the healthcare sector and focused on developing a strong educational and social networking component. In addition, a number of studies have reported on the activities and challenges associated with specific CLAHRC entities, focusing on a micro level of practice [17-19]. Tensions that work to maintain the KT gap have been shown to include the contrasting researcher and provider cultures, knowledge domains, timelines, and incentive structures [20-26].

Yet, there has been little comparative study of multiple KT entities and their capabilities as they work to translate research knowledge into practice and accommodate the tensions inherent in coordinating research and implementation activities [6,24]. We suggest that this is an important lacuna to fill, as governments are increasingly investing in translational entities and leaders would benefit from exploring the different models in practice to inform investment options and leadership challenges associated with the different models. In particular it is important to examine different ways of organizing research activities that are exploratory in nature and conjoining these with implementation activities that focus on the exploitation of knowledge.

In this paper we draw on a comparative case study of nine KT entities in the development of five KT archetypes. Our cross-sectional comparative study of nine CLAHRCs provides insight into diverse organizing logics that enable KT. We highlight, in particular, the coordination and capacity development of exploratory and exploitative dimensions of the KT entities. In the following section we review the health service research literature on the challenges associated with KT. Further, we develop insights from the innovation literature that examines knowledge exploration associated with research activity, and knowledge exploitation associated with implementing existing knowledge to increase knowledge application and organizational effectiveness.

### Challenges of implementing KT

The complexity and challenge inherent in KT is widely acknowledged [5,7,27,28]. As such, a number of distinct ways to accomplish KT have proliferated, including approaches that seek to render knowledge more explicit in terms of systematic synthesis and guidelines, improving social interaction and sustaining relationships between researchers and decision makers as well as emphasizing organizational readiness and contextual features associated with KT [29]. Entities explicitly developed to facilitate KT activity may draw on all these approaches to achieve knowledge utilization.

An important underpinning challenge in enabling KT stems from the knowledge boundaries between stakeholder groups; knowledge boundaries that may be founded in contrasting meanings ascribed to particular knowledge claims, or more fundamental political differences in priorities held by the groups involved [25,30,31]. Managing knowledge flows across these diverse boundaries is difficult, whether the context surrounding the anticipated translation is to enhance the uptake of research [32], new product innovation [31,33] or multidisciplinary collaboration [34]. As recently summarized [35], a number of strategies have been developed to help leaders facilitate the translation of knowledge across stakeholder boundaries, including the use of knowledge brokers (*e.g.*, opinion leaders), boundary spanners (*e.g.*, practitioner researchers), and systematically integrative boundary activities (*e.g.*, regular engagement events) [24-26,35].

Three critical capabilities need to be enabled and sustained by KT entities such as CLAHRC’s, namely: improving research capacity among service providers; producing relevant research findings; and changing service provision in accordance with research knowledge [6,21,24]. The first KT capability relates to the difficulty for practitioners to understand and assimilate the technical complexity of research and their need to develop research literacy so that the new knowledge can be absorbed. The KT term ‘absorptive capacity’ [36,37] has been increasingly used in the health service literature [38] to emphasize the need for examining, appraising and assimilating. *E.g.,* among paediatric occupational therapists, Lyons *et al.* found that frontline staff held positive attitudes towards research and were willing to access research information, but lacked confidence in doing so [39]. Engaging more clinicians and service providers in the production of research has been suggested as an important way of increasing research absorptive capacity, as practitioners become more familiar with research methods, language, and interpretation [40] and can inform researchers on strategies for rendering their findings accessible.

The second KT capability is producing research. It is, however, important that research findings and outputs are relevant to service providers; the seeming irrelevance of the research questions studied has been cited as a reason for low research utilization [40,41]. Participation of practitioners in the research process can make the research questions more relevant and grounded in current concerns [42]. Sustaining the interest of service providers is hindered by the length of time required for conducting research and in making conclusive findings [17]. The co-creation of research between academics and service provider staff points towards the importance of changing the culture of research to be more inclusive. Research within CLAHRCs, *e.g.,* explicitly acknowledges the importance of active participation of different stakeholders in the research process [5,17,24,43,44], which represents a shift away from a passive view of non-researcher participants such as service providers [45]. An important focus and concern has been how lay service users and patient representatives might adequately participate in the knowledge generation [20,43]. Given the historical autonomy held by academics over the research process, and the incentive structure of the academic tenure system that hinges foremost on high impact publications, it is difficult to impose significant changes on the research process, which can alienate elite and highly-qualified academics. On the other hand, in order to legitimize this new area of translational applied research as being of high standard and worthy of publication, it is important to engage top researchers and secure their ownership in the process.

The third capability associated with KT is being able to sustain behavior changes in service delivery for prolonged periods, until the new practices become implemented [46]. As emphasized in the abovementioned capability, making research more relevant by engaging more stakeholders with the research question and the research process can be an important means to facilitate and support adoption and behavior change at the level of service delivery [24,38]. Further, these processes of engagement with service leaders or local champions are important [47] in developing long-term commitment and relationships, which plays an important role in embedding sustained behavior changes [48-51]. Further, a stream of implementation research has foregrounded the importance of considering local contexts to promote sustained changes. *E.g.,* Wensing *et al.*[52] argue for the importance of tailoring new research knowledge for specific contexts to enable implementation. They suggest that systematic tailoring entails three key steps: identification of the determinants of healthcare practice (that is, those factors that might prevent or enable improvement), designing implementation interventions appropriate to the determinants, and application and assessment of implementation interventions that are tailored to the identified determinants.

### Understanding KT challenges through exploration and exploitation

Consistent with the renewed UK government focus on innovation [53,54], we explicitly link KT activities in applied health research with the process of innovation. We draw specifically on key concepts of the innovation literature, namely exploration and exploitation, to examine diverse ways of organizing KT as well as a third concept, ambidexterity, which refers to achieving a balance between exploration and exploitation.

In essence, exploration underpins the knowledge generation processes of health research—and thus doing research—while exploitation underpins service improvement and implementation activities, being explicitly concerned with applying new knowledge to change current practices [55]. These two dimensions of the innovation process can provide further insight into the tensions and challenges of leveraging research to deliver successful improvements in health service delivery, thereby achieving ambidextrous balance between creating and using knowledge. March [55] suggests that the essence of exploration is experimentation to develop radically new ideas while exploitation is centered on refining and extending existing competencies and technologies for more proximate gain. Kang *et al.*[56] elaborate that exploratory processes, which necessarily involve knowledge creation and thus includes research processes, are needed in order to find new and better ways of providing services or new products. New knowledge is necessarily characterized as having initial uncertain relevance, as the outcome of doing research or developing new products is by definition not known; the product may fail or the research may be inconclusive. Yet, it offers high potential for benefits, though at the expense of high costs in terms of its generation. Explorative activity is supported by an ability to value, understand, and apply new external knowledge, commonly referred to as absorptive capacity [57], a term now increasingly used in public health contexts [36-38]. As new ideas and forms of knowledge are combined, tested, and developed, novel findings emerge, *e.g.,* as is typical of research outputs. Innovation research has shown that high levels of external control negatively impacts on innovation outputs, as creativity and novelty are best supported by open processes and high levels of worker autonomy [58,59].

Exploitative innovation processes focus on implementing and refining existing knowledge and practices—such as using a new product, device, or technology. Exploitative innovation can also include implementing a new pathway or process that has been shown to be effective elsewhere into a novel context—*e.g.,* a new user registration system or new tracking process for medicines previously used in another organization. Exploitative innovation has been associated with quality improvement techniques [59] and increased levels of centralized control [60] as they seek to minimize variation and obtain alignment across an organization domain. Exploitative processes are more certain due to their incremental nature thus resulting in more certain outcomes—*e.g.,* when it is already known that the tracking device or new medicine works based on implementation elsewhere, or published reports. While the scope for benefits is limited—*e.g.,* new patents will not be produced and radically new ideas are not anticipated—costs are more predictable. March [61] notes, ‘exploiting interesting ideas often thrives on commitment more than thoughtfulness, narrowness more than breadth, cohesiveness more than openness’.

A challenge frequently highlighted in the innovation literature is the competing demands entailed in organizing for both explorative and exploitative learning as these activities are fundamentally different, requiring different coordination mechanisms, levels of control, and resourcing [62]. One way of organizing for innovation is to develop organizations or collaborations that are ambidextrous—able to perform both types of activities simultaneously [59]. An alternative mode of organizing for explorative and exploitative activities is to create a balance by alternating periods of exploitation and exploration [62]. This mitigates the need to balance the coordination between both types of activities. Through these alternating periods implementation teams, *e.g.,* can focus on delivering exploitative innovation through continuous process improvements, reducing variability and increased efficiencies and control [59].

KT, as a general process of transferring research into practice, can be regarded as constituting both explorative innovation found in research domains and the more exploitative innovation in the implementation domains. Absorptive capacity [37,57], which we argued earlier was a core capability sought after by KT organizations in general (as well as CLAHRC entities in particular), is a relevant and important concept in understanding both exploration and exploitation. The exploration activities of researchers are reliant on the ability to recognize and assimilate new knowledge, but ultimately they need to be directed at application as an outcome of a KT entity’s operation, resulting in behavior changes at service delivery levels. As pointed out previously, in order to engage top researchers, it is important to understand the impact of constraining the autonomy of exploratory processes to the extent that the research is no longer of interest. Given the often competing timeframes of research production and immediate service provision demands, research within what is sometimes referred to as a KT collaborative is generally pre-designated as ‘applied health research’ and may be designed to be less exploratory or radical than other health-related research, such as laboratory medicine. On the other hand, while service providers may concentrate their activities in exploiting research for more practical application, they also have to ensure that they are able to, when needed, recognize and draw on relevant knowledge bases generated by research, which requires absorptive capacity. Through increased participation in explorative research processes, practitioners can engage in the production of research aiding their understanding of research outputs and contributing to the relevance of research questions. Whereas researchers, through exploitative dimensions of innovation, are able to consider the ways in which the research can be used in service provider practice.

We draw on the role of exploration and exploitation in the innovation and KT processes to compare and contrast CLAHRC KT entities. We address the following research question: ‘What are the ambidextrous strategies employed by the CLAHRC entities in translating knowledge from research into practice?’ Our comparative analysis unpacks different archetypes that characterize how CLAHRCs sought to balance the competing demands of exploration and exploitation, how these organizational forms differed in structure, and the nature of innovation they enabled. In presenting our findings of our five archetypes, we discuss the implications for translating knowledge across the research to practice ‘gap’ and conclude with practical suggestions for leaders engaged in KT activities. First, we present our research methods.

## Methods

CLAHRCs were set up in 2008 in England as a pilot program by the Department of Health (DoH) to bridge the research to practice knowledge gap. Each new partnership was given flexibility as to how to organize, with very little guidance from the DoH. The primary reporting requirement was an annual document that detailed the outputs and impact of the respective CLARHC. CLAHRCs organized themselves into research and implementation themes with designated theme leads overseeing the projects; they developed governance mechanisms that enabled organizations to bridge and coordinate the efforts of academics across different departments, and provider organizations. Most of the partnerships involved more than one university department, as well as health (*e.g.*, NHS) and social service providers and other stakeholder groups (such as business sector or public and patient representatives). Each of the CLAHRCs developed novel approaches to bridging the research to practice gap in this context of a natural ‘experiment’.

The research team obtained ethics approval from each of the NHS provider organizations involved in the CLAHRCs as well as each of the directors of the KT entities. Some members of the research team were also involved in a specific KT entity, which further attuned the fieldworkers to the salient issues, challenges and remit of the CLAHRCs. The NIHR Service Delivery Organisation (SDO) funded project was explicitly not seeking to rank the CLAHRCs or their respective outputs, but rather to provide feedback on the overall CLAHRC process in a comparative manner.

The study is based on the qualitative analysis of 106 semi-structured in-depth interviews with the directors, theme leads and managers, key professionals involved in research and implementation from all nine CLAHRCs [9,63]. Most of the interviews were conducted in person, with a small number being carried out over the phone. Data were also collected from intensive focus group type workshops conducted with the key members of five CLAHRCs. Focus group workshops were conducted in collaboration with the colleagues from the RAND Corporation^a^; each workshop included between eight and 23 participants. Interviews and workshops generally lasted for one and three hours respectively. They were digitally recorded and transcribed by a professional transcription service. Researchers also attended a number of meetings in association with CLAHRC organizations, including seven theme leaders’ meetings in three different CLAHRCs, one CLAHRC directors’ meeting, four CLAHRC cross theme learning activities, three feedback sessions, and three CLAHRC-wide events organized by the central funding agency. During these meetings and events, detailed notes were taking regarding challenges, CLAHRC structure, KT processes, and perceived leadership challenges.

The interview protocol included questions focusing on the goals of the CLAHRC; process and mechanisms behind their adoption; organization of the theme or research projects; vision for KT; activities (*e.g.*, projects, collaborations) used to attain KT goals; key challenges encountered; breadth of stakeholder involvement across the spectrum of activities; and relationships between research and implementation themes. The interview protocol was also adapted to the specific expertise of an interviewee. For example, senior managers (*e.g.*, directors, deputy directors) were able to provide a more generalized understanding of the goals and means of the CLAHRC. In comparison, representatives of research implementation themes provided a more nuanced understanding of the practical challenges faced in the execution of KT goals or networking patterns across research and implementation themes.

Data analysis and KT model development occurred in three stages. In the first stage, data from the first 52 interviews—approximately six from each CLAHRC conducted in the second year of operation—was qualitatively analyzed and coded using Atlas.ti. This yielded broad themes of similarities and differences in approaches used by CLAHRCs in developing their early KT vision and coordination across partnerships. Themes were further refined by using tables and graphs to display and organize findings [64]. We then re-analyzed the transcripts for perceptions regarding the strengths and weaknesses of KT within and between CLAHRCs and sought to link these to the differing approaches used to coordinate KT activity. Because our focus was a cross-sectional comparison across CLAHRC entities, we did not emphasize the ongoing changes in structure as CLAHRC entities learned from each other during later stages of CLAHRC programs.

In the second stage, we developed written narratives of contrasting KT models, highlighting the strengths and challenges associated with each. We used these discursive scripts to engage and discuss with at least one senior member from each of the CLAHRCs regarding their perceptions of their CLAHRC KT model and get feedback on our insights. From this we developed schematic representations of archetypes for organizing KT activities found within and across the CLAHRCs. We then went back through the further set of uncoded 54 interview transcripts—conducted in the third and fourth year of operation—as well as focus group meeting transcripts to examine the fit of the typologies with the data.

In the third stage of analysis, we further developed the validity of our schematic diagrams by presenting our findings to three separate groups of CLAHRC stakeholders. Two groups represented eight to ten senior stakeholders, including the directors and deputy directors, involved in one respective CLAHRC who were given the five models as part of a formative evaluation process of their own KT approach (in consideration of their reapplication bid). The third group was more eclectic, incorporating 80 individuals representing all nine CLAHRCs and who spanned across the hierarchy in terms of seniority. The schematic models were presented in a workshop format to solicit feedback on archetypes from the diverse stakeholders. Schematic models were then further refined using the feedback from these groups. While there was a strong sense in which the models reflected the CLAHRC organizing structure, a key point of feedback was regarding how patient and public involvement fit into the respective models^b^. Importantly, we highlight in this final stage that the models are not representative of all the characteristics of one CLAHRC partnership, but rather a synthesis of distinctive strategies used by CLAHRC entities into an archetype. As has been previously highlighted regarding such ideal archetypes of organizations [65], real organizations seldom, if ever, reflect all the features of an ideal type, but rather different aspects and varying degrees of these features. Rather archetypes are a set of structures and processes that reflect a single interpretive scheme that orders everyday practices [65,66].

## Results

In the following section, we organize our findings around five KT archetypes that became evident in the comparative analysis across CLAHRCs. Though CLAHRCs generally organized their KT approach predominantly around one of the archetypes, CLAHRCs drew on features from several models. Thus, while our descriptions are based on the empirical cases, our purpose is not to delineate the relationship of particular CLAHRCs to specific archetypes, but rather to reveal the breadth of KT approaches that developed in this ‘natural experiment’ of a unified context, unified goals, and where organizational approaches emerged independently. A number of similarities were evident across all CLAHRCs. Key similarities that featured across multiple levels and stakeholders within CLAHRCs were the temporal challenge of integrating research and implementation activities, including the timeframe of managing the ethics process, different priorities of academic and provider organizations, measuring empirical impact, perceived difficulties in publishing highly contextualized research, on-going health and social care reorganizations, and finding adequate means of integrating patient and public concerns into research and implementation processes.

### Archetype A: involving a broad array of stakeholders in a multidisciplinary research process

**Figure 1:**
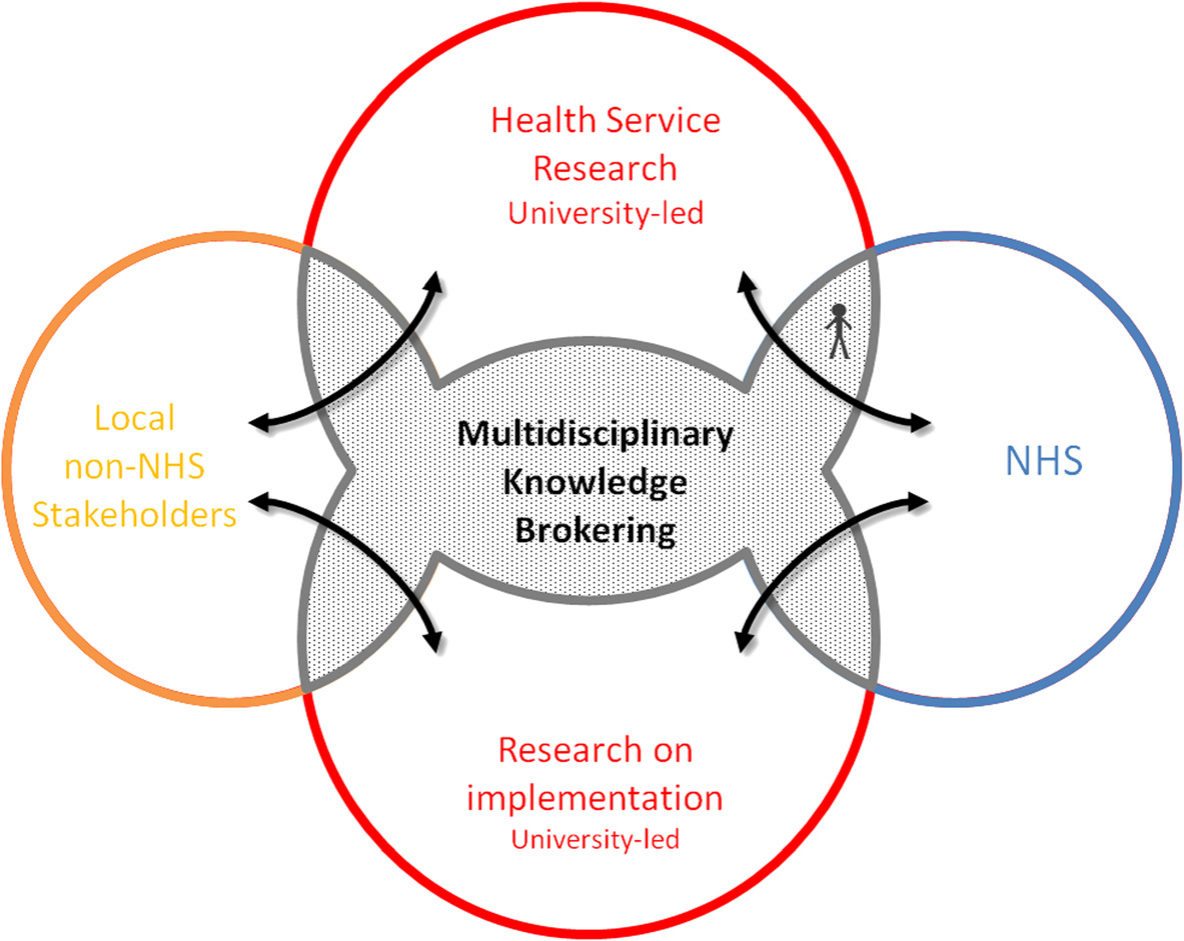
Archetype A: Multidisciplinary research.

One way to organize KT activities entailed the purposeful integration of multiple stakeholder groups into the research process, so as to address research questions concerning highly complex problems from novel perspectives. This could include researchers from multiple academic backgrounds, patients and service users, as well as practitioners and managers from diverse organizations—see Table 1 and Figure 1 for a diagrammatic representation. KT entities organized around Archetype A maintained exploration as a focus but with a new inclusive culture of research being enabled as new stakeholders, such as patients or health service providers, became involved in research supported by a knowledge-brokering process. The expanded groups of stakeholders became involved in designing the research question, collecting data and receiving on-going feedback on research progress and early findings, restructuring in some part how explorative research was conducted to make it more relevant to implementation. An important emphasis in this approach, therefore, is to alter the culture of research from uni-disciplinary silo activity to accommodate more diversity; an aim is to improve research relevance, as well as gain broader ownership, enabling the knowledge produced to be more easily exploited ‘downstream,’ and promote ambidexterity. Research activity in this context focused on complex multifaceted problems that by their nature require multiple perspectives to address. This archetype emphasizes rigor in the research process but in doing so, seeks to adapt the potential relevance of the research output as well as widen the stakeholder ownership among entities who may subsequently exploit findings in healthcare delivery^c^. From the perspective of the researchers and practitioners involved, this model of KT radically alters the process of exploring and generating new knowledge and was seen as uncomfortable because researchers needed to accommodate the advice of non-academic stakeholders (*e.g.*, service providers and practitioner researchers) and include them in the research activities:

‘This is the most radical thing the NIHR has ever done. I have never done research like this before … it is completely different.’ (Academic lead)

‘We used to stand outside and look in at [the research process] but now you have opened up the windows and let us in … [it is great] to participate in the research [process].’ (Senior manager, service provider)

**Table 1:** Archetype A

**KT archetype &organizing logic**	**Explorative dimension**	**Exploitative dimension**	**Strengths**	**Leadership challenges**
Archetype A	Research **governance** maintained by academics yet they are accountable to a wider group of stakeholders; this can increase researcher **absorptive capacity** of service provider values and concerns.	**Exploitation supported** by seeking to shift the culture of research to integrate broader set of perspectives and stakeholders.	Increased stakeholder involvement enables integration of perspectives, thus suited to researching complex multidimensional problems.	Complexity of research and integration of (shifting) stakeholder agendas can increase the time needed to generate research outputs.
Multi-stakeholder research to engage a wide range of perspectives	High exploratory focus maintains **academic autonomy**.	Wider research engagement enables research to be more relevant to users and increases their **absorptive capacity**, being more aware of research process.	Research includes the KT process, which may be done from multiple perspectives.	Brokering and negotiation needed across multiple stakeholder groups.
	Wider research agenda promotes research into implementation processes from multiple perspectives.	Engaging practitioners and health service providers in research increases their level of ownership, supporting the implementation of research findings; yet implementation process not formally controlled.	New culture of inclusive and multidisciplinary research can generate wider genre of research, beyond medical paradigm.	Risk of alienation and retreat to institutionalized silos of activity if boundaries are not actively managed, rather than sustaining new culture of multi-stakeholder research.

In the context of the CLAHRCs, this model also emphasizes doing research on KT as a process and science of implementation, including how patients and public might be involved in knowledge generation^d^; implementation themes may draw on the multiple stakeholder perspective to explore the dynamics of implementation itself. Thus, a core output of this model is to generate conceptual and systematic knowledge regarding implementation and KT and ultimately facilitate exploitation practices:

‘Our approach in the implementation themes is not about going into the hospitals and telling the nurses and doctors what to do, but to engage with these and other stakeholders to understand implementation [challenges] better. We are academics … we don’t have [the] jurisdiction to go into hospitals or social services and tell them what to do. But we can develop knowledge that will help facilitate the process.’ (Implementation Theme lead)

A strength of this KT model is its lack of compromise on the rigor of research as researchers retain high levels of autonomy over the exploratory process, with the potential for the new knowledge to be radically innovative given its multidisciplinary breadth, as well as its potential to address complex problems in a systematic manner:

‘Ours is very research orientated, it’s very, very much so… ours is very academic based which is good; it just takes a bit longer to get things going… But I think in terms of sustainability the way we do it is much more sustainable because we are producing the evidence that should be sustainable.’ (Director)

‘A strength of our CLAHRC is its [multidisciplinarity]….*e.g.*, our business school does research that develops the science of moving knowledge around a system.’ (Director)

Allowing high levels of researcher autonomy can enable research teams to adjust and renegotiate their projects to fit with the needs and emergent context of stakeholders involved; however, this level of co-production is dependent on adequate flexibility in the exploratory research design as well as researchers’ mind set. Sustaining broad research engagement and stakeholder facilitation is an important role of the central management.

Organizing KT activity in this manner relies on high levels of collaboration and mutuality; breakdown across stakeholder boundaries is difficult to manage and liable to make existing groups retreat back into silos and comfort zones of historical relationships (of non-integration). To maintain a generative exploratory KT process with ongoing co-production, central management leaders would need to maintain loose coupling across the diverse agendas and not be seen to favour one stakeholder group above another, to avoid alienation and return to compartmentalization:

‘In principle collaborating across departments is a great idea. But we have issues of data ownership, which type of research publications to focus on, and how to work together…. In the first two years we used to work more closely with other themes … but it is hard… and now we are farther apart. I notice this across the board that sustained engagement is [difficult].’ (Theme lead)

Our analysis found fundamentally sustained changes associated with widening the stakeholders engaged with the knowledge exploration; the successful implementation of this model is associated with the nature of the research process itself becoming more inclusive and using multiple perspectives in ongoing co-production, including both social science and medical worldviews. Exploitative functions are enabled through ownership by way of co-production and general increases in provider absorptive capacity through participation in research activities. The breadth of stakeholder perspectives accommodated within the research process also enables exploitation because the research questions are more relevant to current provider concerns and take account of the complexity of service delivery contexts.

### Archetype B: loosely autonomous research streams with designated knowledge brokers

**Figure 2:**
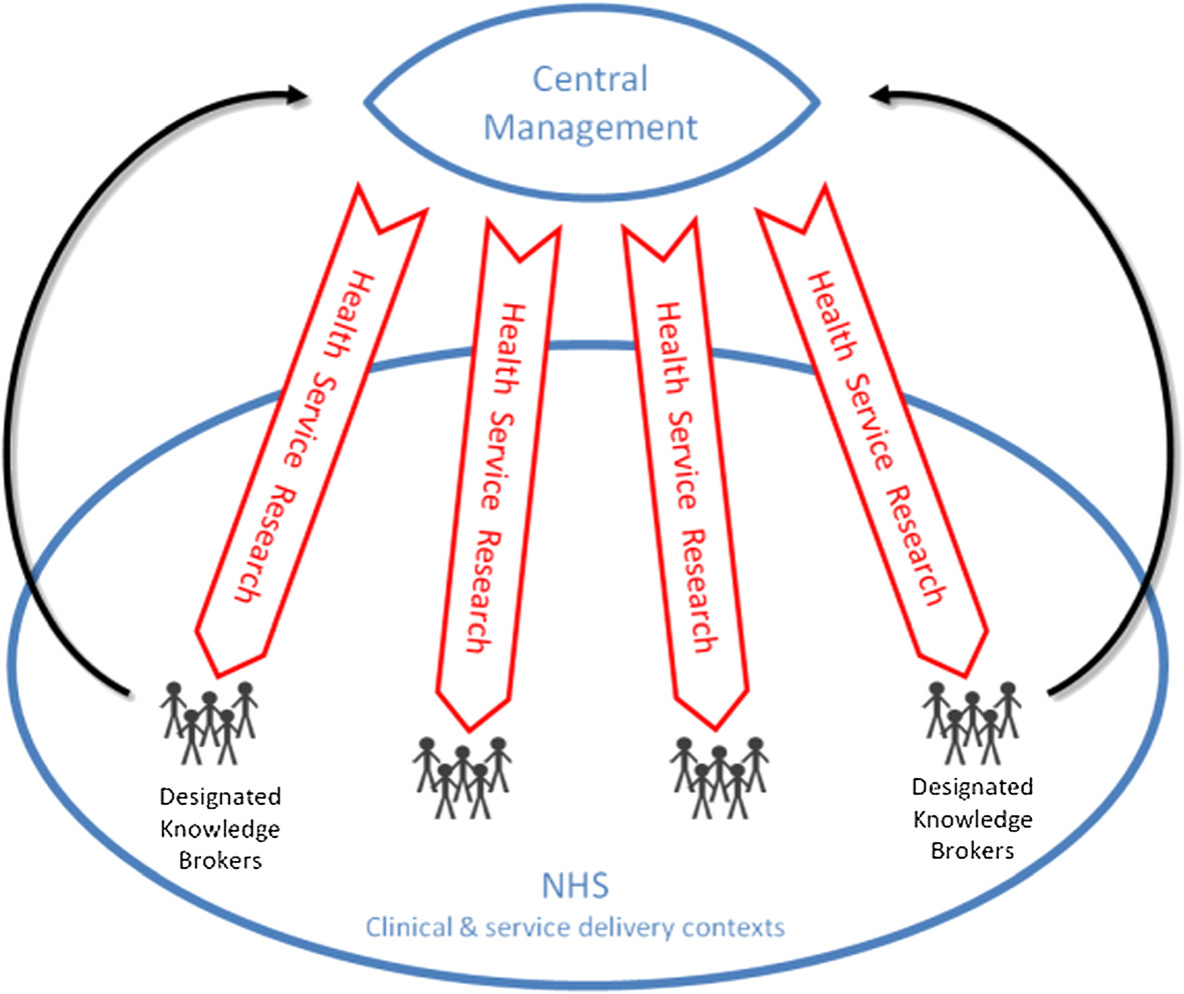
Archetype B: Designated knowledge brokers.

KT activity can also be organized around loosely structured collaborative research projects that have a number of designated knowledge brokers attached to each project—see Table 2 and Figure 2 for a diagrammatic representation. The emphasis is on structuring the implementation processes within specific service providers. Explorative research activities remain largely unchanged, although they accommodate brokering agents who contribute contextual insight and retain ownership of the exploitation activities. Knowledge brokering agents have the responsibility to exploit the knowledge being developed by researchers. As knowledge brokers work across two distinct stakeholder groups, having managers who are familiar with frontline care delivery and thus implementation issues seconded to work within the CLAHRC for a designated portion of their week enables dialogue and integration of explorative and exploitative activities promoting ambidexterity. In this way, research activity can be organized and controlled by university researchers^e^, yet the research questions can be negotiated with the designated knowledge brokers, who have critical knowledge of provider issues and have established relationships with the research team. Balance between exploration and exploitation can be maintained, supporting both research and implementation—see Table 2:

‘[My role was about] ensuring that the projects … linked into the NHS through [designated brokers] and things like that and the engagement theme was about engaging with wide stakeholders like members of the public and patients.’ (Implementation theme lead)

**Table 2:** Archetype B

**KT archetype and organizing logic**	**Explorative dimension**	**Exploitative dimension**	**Strengths**	**Leadership challenges**
Archetype B	Research **governance** maintained by academics, including process and questions; yet select KBs are invited to interact with research teams.	**Exploitation supported by** KBs in accordance to KB jurisdiction.	Research capacity within service providers is developed through KBs.	Difficulty appointing KBs at the right level of seniority to effect and resource service change.
Designated knowledge brokers (KBs)	High exploratory focus maintains **academic autonomy**.	**Central management** organize and support KBs.	Researchers can develop sustained dialogue with provider representatives to facilitate on-going relationship following project completion.	There is a risk researchers can focus on exploration and disregard concerns of service orientated KBs and knowledge exploitation, given no formal accountability between academics and service providers.
		KBs receive formal training in brokering techniques and skills, increasing individual and system level **absorptive capacity** to draw on research knowledge to influence service delivery.		
	KBs are aware of research agenda and nature of likely findings, thus able to develop implementation goals early in research process.	KBs responsible for embedding findings in local services, being **accountable** to local services.	Designated KBs have ownership for supporting KT into specific service contexts.	

The ideal type of this KT model requires brokers with varying levels of time commitment and seniority within provider organizations, to balance their ability to broker with relevant target audiences across the spectrum of provider services:

‘We are trying to fully integrate [knowledge brokers] into our [research] team so that they know what stage we are at with the research. They helped write the implementation component for each of our proposals and we also have used an implementation contact to work alongside our [knowledge broker], so they do a bit of education and teaching and support with the [knowledge broker].’ (Theme leader)

As each provider organization has a unique context and each research theme have their own particular dynamics, having designated individuals charged with brokering the boundary across these domains allows them to develop locally suitable ways of integrating KT processes. Individuals can work to their strengths and nest activities into the unique context^f^, making the new boundary dynamics sustainable through long-term relationships. The implementation activities of brokers, helps create conduits between the diverse groups thus bringing synergy across the different projects.

An important feature of this model is its potential to develop absorptive capacity within provider organizations. Knowledge brokers can be trained and given skills in interpreting research methodologies and managing change processes within their organization. Training programs designed by central management integrate across diverse stakeholder worldviews and can be tailored to the profile of provider organizations and participant experience:

‘The dissemination team committee which meets regularly, … the committee [is] trying to run all these … classes so economics classes e.g., [and] sociology of networks for the [knowledge brokers].’ (senior manager)

Importantly, as the designated brokers and boundary spanners originate from provider, rather than researcher, organizations, they retain ownership of the exploitation process. Given the designated brokers’ breadth of contacts, they can access and draw in a range of resources if they have sufficient status within their host organization:

‘[Designated brokers] are located in the NHS and are designed to link clinical and/or managerial problems into CLAHRCs so the CLAHRC can offer expertise and assistance and help doing either research or doing some of the knowledge generation that isn’t research to help them address that. So it is a way of ensuring that ownership of some of these issues remains with the NHS.’ (Senior manager)

Yet an ongoing challenge in the KT model is the tension inherent in integrating traditional research activities with ongoing engagement with stakeholders so as to facilitate exploitation. Researchers are likely to divert their attention and interest in knowledge exploitation activities towards the production of interesting and publishable findings:

‘There is this continuing tension as you probably know between them running off with their projects and the rest of us saying ‘hang on, this is about knowledge transfer, this is not just about stroke.... but there are tensions because they are the people that are trying to sell dissemination and [knowledge brokering] …and it is quite clear that the PIs and some of the Research Fellows aren’t terribly interested in that.’ (Implementation Lead)

Similarly, in the current fiscal climate of healthcare providers, it remains a challenge for providers to continue releasing the designated knowledge brokers and boundary spanners away from their normal activities. Pressing matters frequently draw them back into their former routines, as the priority of research can pale in the face of current crises:

‘There has been a lot of effort to try and release these [designated knowledge brokers], but other key people have not been released from their time even though you could argue that the Department of Health and the other contributors of funding feel that they have been charged for it.’ (Research theme lead)

### Archetype C: independent research and implementation activities

**Figure 3:**
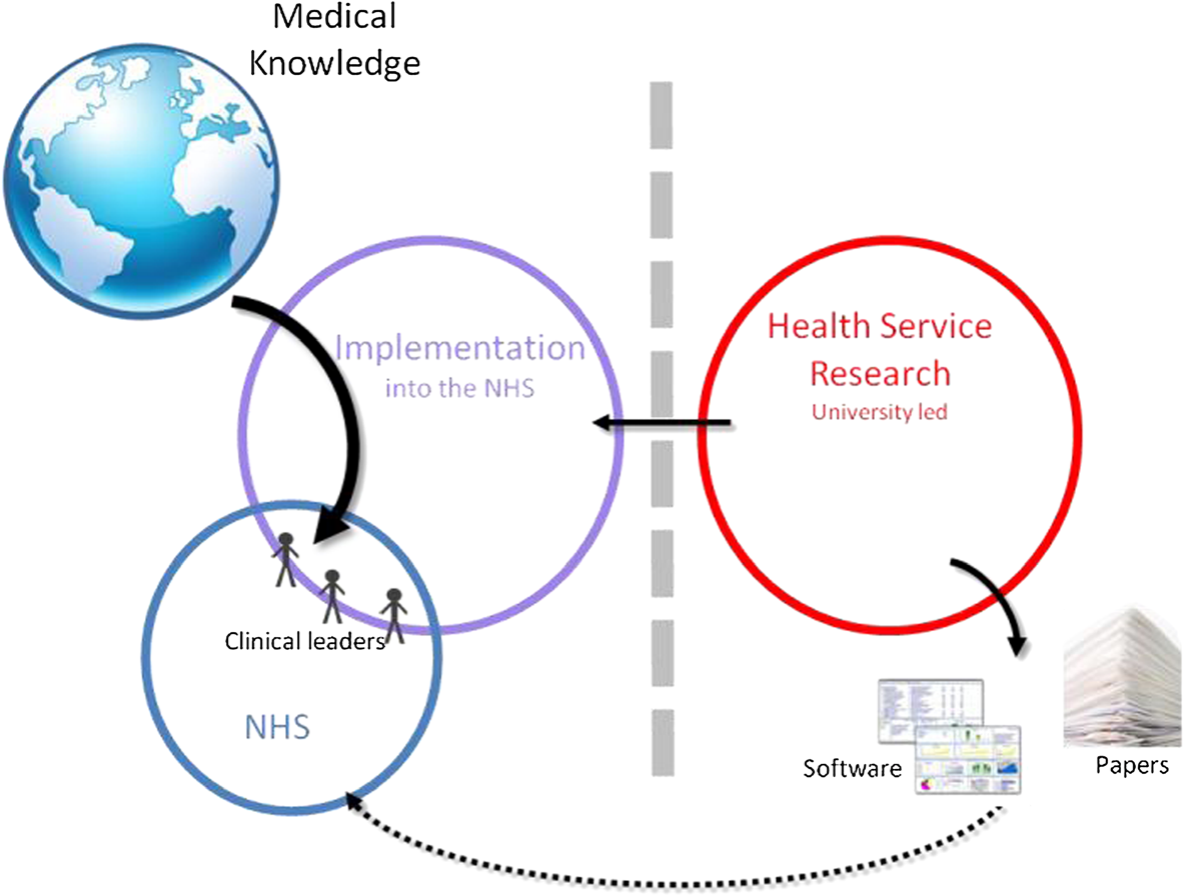
Archetype C: Modular independence.

Another typology for organizing KT activity entailed the separation of research and implementation activities, maintaining them in parallel as independent modular functions—see Table 3 and Figure 3 for a diagrammatic representation. Ambidexterity is achieved through separation rather than integration. Given the distinct temporal dynamics of research and implementation activities, non-integration enables focus on exploratory research activities by academic researchers working on topics they choose and know can be published. The research themes may broaden their concerns to consider the practical relevance of their work, but this is not their focus. Patient and public involvement can be incorporated within the research process as per existing guidance available from the central funding agency, but is likely to be more problematic to incorporate within the implementation processes as few standards exist to guide their involvement and the processes are kept separate:

‘We are not trying to do research in a different way fundamentally, I mean the mechanics of research, but we want the research to be more aware of some of the contextual issues that then tend to change people’s thinking about how they do it.’ (Deputy Director)

**Table 3:** Archetype C

**KT archetype and organizing logic**	**Explorative dimension**	**Exploitative dimension**	**Strengths**	**Leadership challenges**
Archetype C	**Research governance** maintained by academics who determine research questions and process.	**Exploitation supported** by development of implementation skills, as organized by **central management**.	Quick start to implementation process as not waiting for new research findings to be produced.	Boundary between implementation and research themes, stymieing integration between their efforts.
Modular independence	Highly autonomous exploration with no explicit need to accommodate new significant stakeholder groups.	Existing external knowledge used, such as systematic reviews and other published accounts of research outputs.	Autonomous research process attractive to highly qualified academics who are not needing to change their research practice; this increases likelihood of high impact generalizable findings.	Low co-production of research topic risks knowledge outputs having low relevance to local stakeholders.
	Exploratory focus maintains **academic autonomy**.	No explicit link with in-house research process.		
		Increases **absorptive capacity** through acquired skills in identifying and appraising research evidence and reviews.		

Implementation activities focus on exploiting all existing health research available in published literature. Rather than integrating with exploratory knowledge production activities, this modular organization of parallel activity develops service providers’ knowledge regarding relevant evidence already published. Given the wealth of well-established research and best practice guidelines, this model of implementation is efficient since it incorporates an already available corpus of research knowledge into service improvement processes rather than spending resources and time producing more knowledge^g^. Hence service improvement is primarily informed by searching for already existing external knowledge rather than creating new knowledge internally:

‘Other CLAHRCs have these very clear things, you know, … teach management to researchers … plus the implementation guys understanding how those components actually go together to create a situation where the two can speak to each other. We have not done that in any form.’ (Director)

An important advantage of this KT model is the speed by which implementation activities can ramp up. Rather than waiting for the end of a lengthy research study, change agents can develop local capacity within service providers and influence practice more quickly using existing knowledge outputs. Once networks are established to influence health service practice, these can later be used to mobilize findings from the organizations’ own research program:

‘The implementation people having got such a head of steam and worked hard to create this momentum and movement for change, they don’t want to be checked in the process by research slowing them down.’ (Deputy Director)

The slow temporal rhythm of research, as compared to service provider environments was a consistent cause of strain across all CLAHRCs; while research production and knowledge creation is a slow and meticulous process, NHS clinicians and managers were being confronted daily with making decisions in contexts where sufficient information was elusive. By separating the implementation activity in a modular fashion, focus could be kept on exploiting relevant knowledge and evidence from existing research in a co-production manner focused on current provider concerns. Supported by central management, this implementation activity entailed building research literacy among service providers, teaching them sustainable ways of finding relevant research to address their current needs, an important means of developing absorptive capacity.

This model enables a focus on research rigor, through the maintenance of researcher autonomy, ensuring that new knowledge within the funding constraints of ‘applied health research’ is produced and published in a traditional fashion. Given that broad stakeholder involvement and multidisciplinary collaboration is slow and resource intensive [34], a KT model that allows researchers to focus on the complexity of the research task can be more efficient in developing knowledge outputs, as compared to Archetype A; Archetype C may be particularly relevant for straight forward clinical questions that require less contextual embedding so as to enable eventual exploitation:

‘As a research team our goals are very much incentivized by the researchers, universities and the REF. Working here you cannot escape your targets … If you don’t publish then forget it. So 90% of our effort has been making sure that our findings are published.’ (Researcher)

The activities of the implementation themes can be closely coordinated with the current priorities and knowledge needs of provider organizations. Given the novelty of CLAHRC entities and the ambiguity of their role in relation to provider organizations, closely aligning the implementation process with provider priorities was an important means of gaining stakeholder commitment and support.

Yet the limited relationship that develops between research and implementation theme activities remains. While the intention might be for exploratory research themes to draw on the exploitation expertise of implementation themes to disseminate their eventual outputs, this exchange was difficult accomplish in practice:

‘I don’t think there is a model of knowledge transfer [between research and implementation themes]. I think we know that we are not doing a very well on that… I don’t think over the three or four years that we have been together [that] we have quite understood each other and we have certainly not worked together.’ (Theme lead)

### Archetype D: collaborating through loose networks

**Figure 4:**
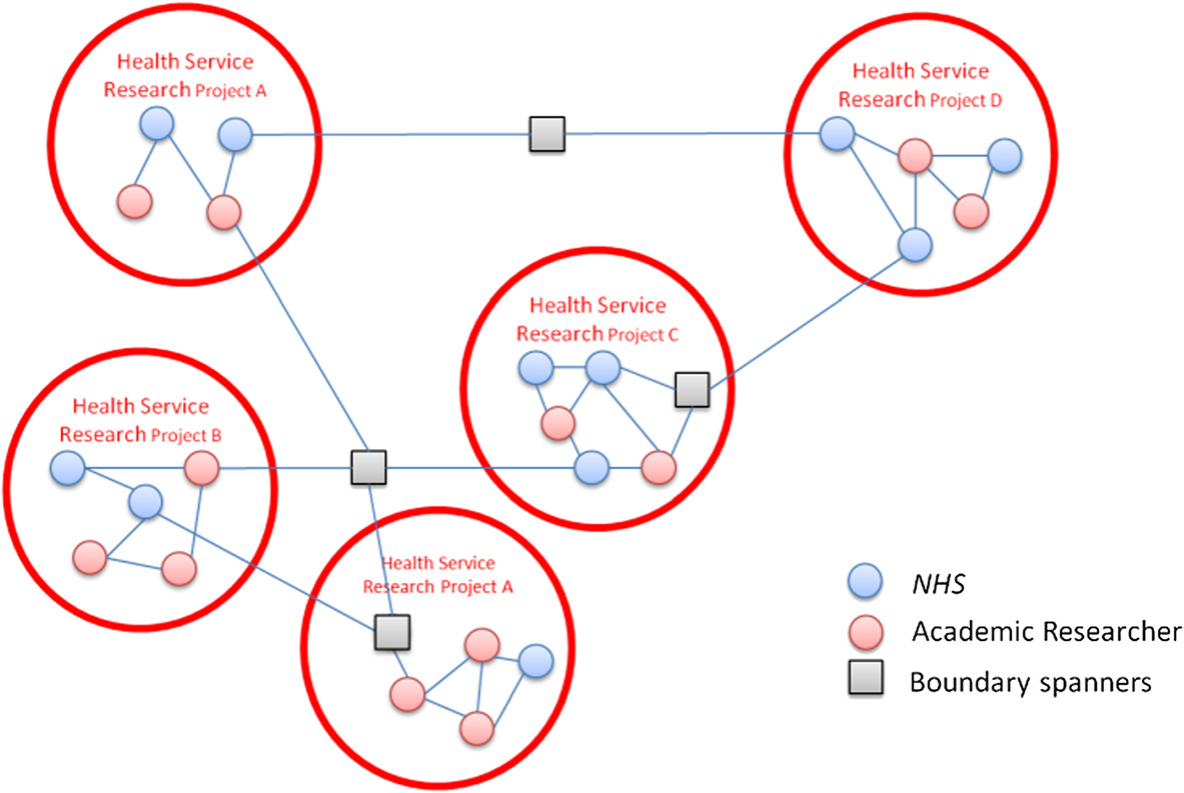
Archetype D: Building on existing networks.

Organizing logic of KT activity in this archetype is through loosely coupled and regionally embedded networks as shown in Table 4 and Figure 4. This model of KT takes advantage of existing informal structures and builds organically from existing relationships between service providers, researchers and other stakeholders. This emphasis is particularly important in a context such as medicine where a number of academics are also practicing clinicians in provider organizations, with existing social ties to draw upon. Recognizing the knowledge exploration and exploitation activity occurs in a context where existing collaborations are already taking place, this model seeks foremost to develop and extend current organic activity in pursing ambidexterity:

‘Much of the [early CLAHRC] work was done by the informal collaborations that we already had going. There would often be clinicians, academics and so on working together.’ (Director)

‘There was an historical context within which we began that was partly because it is a relatively small patch and so there were a lot of personal relationships that had already been established.’ (Director)

**Table 4:** Archetype D

**KT archetype and organizing logic**	**Explorative dimension**	**Exploitative dimension**	**Strengths**	**Leadership challenges**
Archetype D	**Governance** of research process shared between academics and service providers.	**Exploitation supported** high levels of trust that facilitate emergent connections between research and implementation.	Low levels of inertia to overcome at early stages, as individuals already have connections and goodwill ties.	Cliques and silos can arise from unconnected groups within network as no designated brokers are accountable or assigned.
Building on existing networks	Academics and service providers involved in research process; existing relationships form the basis for the collaboration, relinquishing some **academic autonomy**.	Efforts to balance research and implementation goals in the early phases are assisted by existing structures and informal mechanism rather than **central management**.	High levels of possible integration and tailoring of research projects with local provider needs.	Informal governance is difficult to hold to account.
	Research questions heavily influenced by local provider concerns.	**Absorptive capacity** enabled by increased practitioner involvement in research.	Strengthening existing ties enables solid basis for legacy to remain once funding for overall initiative ceases.	Difficult to extend the network beyond certain size when working more informally as this is not centrally managed and more *ad hoc*; ICTs can help facilitate this.

This archetype was used in contexts where multiple alliances between researchers and health providers (and other key stakeholders such as industry) were strong and where hierarchy between research and providers was not emphasized. Existing relationships of trust and goodwill can provide a basis for building partnership projects and a means for arbitrating between competing organizational priorities and goals. In this way, governance structures set by central management can be more informal, guided by mutual goodwill. Networked project groups can also incorporate public and patient participation and their respective social networks^h^. Similarly, other stakeholders, such as social services, charities, or industry can also be accommodated within specific project groups, as enabled by project goals and local relationships:

‘To put this another way - we are taking about a collaboration here, and what you are saying is that there was already an extant collaboration in place which you could draw on. … There was this chunk of good will to work from.’ (Director)

A feature of this model is the early efficiency gains that can come from cooperative decision making and existing goodwill. Having established means for working together, existing relations can provide structure for how to organize new projects and set joint goals:

‘This is not about transferring knowledge from those who have it to those who don’t and almost semi-accidentally we have produced a system where because we actively try to find where the problem lies within the system, and it is not about us providing knowledge, it is about us working with the people for whom it is a problem to try and create something together.’ (Deputy Director)

For example, while the challenge of overcoming the multiple hurdles for research governance were highlighted repeatedly by CLAHRC organizations, using this informal network efficiency can enable new projects to initiate quickly. By seeking to organically embed exploration activities within provider concerns, genuine engagement is possible between providers and academic researchers (and other stakeholders), as neither is able to carry out the networked activity without adequate participation and engagement with the other:

‘You are trying to move from the NHS being a passive recipient of, or purchaser of, or commissioner of research, to being a partner in generating knowledge.’ (Director)

As knowledge brokers are organically nurtured, rather than centrally controlled or appointed, an organizational challenge entails the need to further develop brokers who can span between project groups and build scale beyond the initial collaboration level. While research-based institutions such as universities have established structures for scaling and promoting knowledge exploration, embedding exploratory processes within provider organizations can limit scaling processes, particularly within a context of reorganization and austerity. Thus, a network-centric orientation to KT activities relies on knowledge brokers to take initiative:

‘We started with a theory that we needed to have [brokers] within the CLAHRC who were very clearly seen as having their primary label, allegiance, reality as being within the NHS.’ (Director)

Organic development of new project ideas, building new relationships and translating knowledge around the wider health system can be facilitated through established relationships, often in a manner that appeals to the collegial ethos built into academic and medical professionals:

‘We have a relatively open agenda, although there is always a requirement that it needs to be research that is going to be of some applied use to promoting evidence based practice, but the agenda is not pre-digested. We can generate it ourselves and we can generate it from colleagues in the service and that is very refreshing because increasingly, and especially as we said at national level, the research agenda has become quite heavily bureaucratized.’ (Research lead)

Yet, brokers need to be strategically located in order to maximize their network impact and broker across unconnected groups or network entities. In addition, brokers are likely to need strategic placement in order to minimize clique formation or isolated groups in the network. Without adequate brokers organized to enable knowledge flows, it is difficult to strategically scale the network systematically:

‘What has been more difficult, much more difficult, has been to get beyond that network of fifty, sixty, seventy people, to get beyond that.’ (Director)

A challenge to any informal network is the non-hierarchical leadership and governance process, as it remains by definition loosely coupled and implicit. Hence, accountability mechanisms are difficult to monitor, relying heavily on relational trust and goodwill. A summary of these challenges as well as the strengths of Archetype D organizing forms is provided in Table 4 and Figure 4.

### Archetype E: centrally controlled service improvement projects

**Figure 5:**
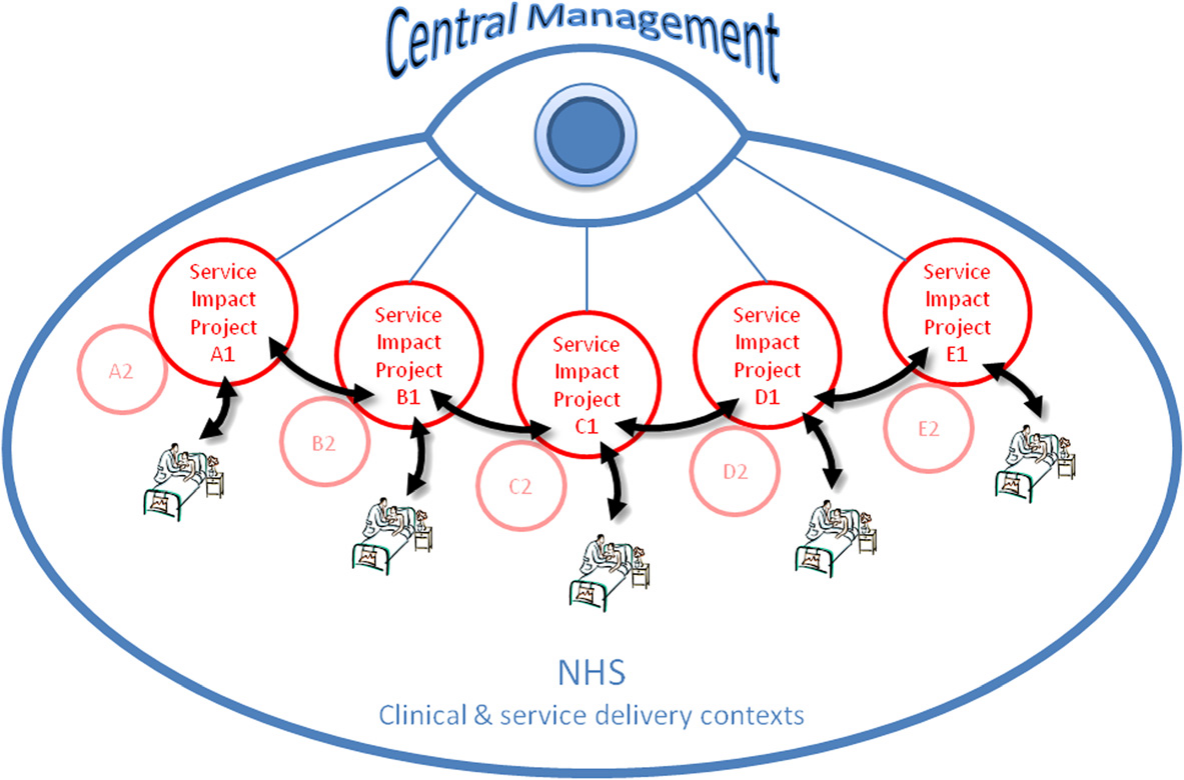
Archetype E: Central management control.

This model of organizing KT, shown in Table 5 and Figure 5, is managerially focused retaining control over both research and implementation activities through ongoing accountability mechanisms and formalized structures to monitor projects. Mechanisms for exercising control include centralized budget management and formal accountability metrics in accordance with central management priorities, which together enable ambidexterity. This model for organizing KT places a very high emphasis on knowledge exploitation for improved service improvement:

‘Well I suppose the guiding philosophy for the whole CLAHRC is that we actually want to make a difference, this isn’t just research, so in terms of what we are about it should be about making it better for patients and then making it better for staff. … If we know we don’t [deliver a service] terribly well, how do we learn from that and change it into something we do do well.’ (Senior manager of CLAHRC)

**Table 5:** Archetype E

**KT archetype and organizing logic**	**Explorative dimension**	**Exploitative dimension**	**Strengths**	**Leadership challenges**
Archetype E	Research explicitly managed by central controls, who hold **governance** oversight.	**Exploitation supported** by systematic approaches to quality management, ensuring consistency.	Project level control by central management enables high levels of accountability.	Low levels of research autonomy risks alienation of high calibre academics.
Central management control	Research directly influenced or determined by local provider concerns, thus low **academic autonomy**.	**Strong central governance** and organization.	Sustained investment in local service improvement.	Incremental nature of service orientated research and alienation of academics decreases likelihood of high impact publications.
	Exploration is more incremental.	High likelihood of research implementation due to **central management** control and highly contextualized improvement- orientated research.	Integrates into culture and goals of a hierarchical health service system.	
	**Central management** systematically collects and collates research findings.	**Absorptive capacity** about implementation processes and service improvement developed.		

One strength of this archetype is the horizontal, systematic approach to knowledge exploitation (*i.e.*, collaborative research design, evaluation, and implementation) embedded at the project level drawing on quality management techniques, and improving absorptive capacity of service improvement techniques. As these projects necessarily involve highly integrated service provider and researcher relationships, this enables learning and knowledge transfer as well as social relationships among project teams^i^. Central management takes responsibility for organizing events, workshops, and regular cross boundary activities in order to promote learning and integration of research knowledge into specific service contexts; this system is developed so research can be consistently exploited:

‘There are a number of sort of management structures that the core team have developed … obviously the projects by themselves are a series of clinical projects, but to be more than a series of clinical projects the CLAHRC core team has developed these vehicles to spread and transfer the knowledge out.’ (Evaluation theme lead)

Having a centralized hierarchy structures project findings and enables accountability for service impact; frequent updates on the status of the work as well as requested research outcomes can be ordered. Rather than letting project teams work autonomously as self-organizing co-production activities, accountability for specified project outcomes is monitored. New interventions can then be recommended by central management, as they control the research process from a distance:

‘Every week we have to feed back our statistics to central management through this database. We need to do this in order to get the funding. So they are always checking on us.’ (Project team member)

‘We are probably slightly different to the other CLAHRCs in that we have sort of direct contact with our projects on a weekly basis … and we can then look at that data to see if it has made a difference.’ (Central manager)

In order for research to fit into predetermined metrics and categories, the new knowledge is limited to being incremental and predicable. Project teams are instrumental in executing project activities and learning, however the overarching plan is held centrally and thus neither locally emergent nor reconfigured without sanction from management:

‘So we do have timelines and we do have accountability and we do have governance, you know, we can change some of those things if there is good evidence that we are doing things wrong, but basically we have a plan and we are trying to work to that plan.’ (Deputy Director)

In order to be exploitative, research projects aim to meet current provider needs, with specified outcomes written in to project deliverables. Instead of researchers exploring areas of interest or novelty, projects are defined in relation to exploiting current service provider concerns. Centralizing the interactions ensures that these are spread out across relevant stakeholders:

‘We did a lot of groundwork by going out to see various chief executives and various other medical directors on the patch in both primary and secondary care… we [asked] a whole variety of people, clinicians and researchers … basically looking for them to give us ideas about what projects to put forward because we wanted things that were on the shelf but also that ultimately fitted our final brief.‘ (Deputy Director)

A high proportion of the project members and event leaders are not research academics, but individuals involved in service provision or part time consultants hired for the job of exploiting research findings and getting change implemented. As commented by one project manager whose role encompassed engaging the new Clinical Commissioning General Practitioner leads:

‘It takes a lot of time, I am out there on the beat, meeting people, calling people… sometimes they don’t show up or cancel. But slowly we are making an impact.’ (Project manager)

As many of those participating in CLAHRC activities are from health service providers, they will be accustomed to the more rigid control and hierarchy culture common in provider organizations and project management teams.

This model of organizing KT is less likely to produce radically novel findings; research-intensive academics may also avoid the seeming instrumental control of management and it may therefore be difficult to attract these researchers to participate. Overall, research outputs are likely to be incremental, according to the transference of research findings from the innovation field on exploitation learning [59,62] and thus at risk of having low levels of publication impact, though local service improvements would be expected to be significant [59,62,67]. While concern for local service improvement is of interest to local providers of that specific service, its highly contextual nature limits the broad generalizability of findings. Hence exploration is highly constrained in order to favour exploitation as summarized in Table 5 and Figure 5.

## Discussion

Our study identified five archetypes of KT that were drawn on by the CLAHRCs. We found that each of these typologies organized KT activity by emphasizing different organizing logics for managing knowledge across the multiple boundaries. Each sought to address the KT gap by coordinating exploration and exploitation activities in distinctive ways and by achieving an ambidextrous balance of these innovation processes.

Organizations are products of their administrative heritage [68], which leads to path-dependency that reinforces existing patterns of behavior [69]. Research organizations therefore, will continue to be fundamentally inclined to focus on exploration activities while organizations involved in implementation will essentially be focused on exploitation. At the same time, however, there are numerous and diverse ways in which the CLAHRCs as KT entities adopted unique strategies in balancing explorative learning within exploitative processes.

KT entities organized around Archetype A maintain exploration as a priority, but with a new inclusive culture of research that engages directly with service provider stakeholders in order to influence the research content and process, while improving its practical relevance. The ambidextrous balance is enhanced through explorative research of implementation processes, which increases absorptive capacity concerning new perspectives in research. Thus, the explorative outputs entail applied health knowledge in areas that are complex and multifaceted, as well as knowledge concerning how the knowledge gap between research and practice might be bridged. The relationships between stakeholders are newly established and relatively weak and unstructured in comparison to other archetypes; as highlighted in the innovation literature [56], these loose connections between previously unconnected groups favors and supports exploratory learning as many new perspectives and stocks of knowledge are combined. A risk of the weaker links between stakeholder groups is the potential for domination by certain groups and the alienation of others, which may lead to groups returning to their institutional silos where strong relationships already exist. Previous research has highlighted the need for structured coordination across diverse communities [19,34], which points to a role for leaders and central management to facilitate brokering and to limit domination of particular groups [70], ensuring that stakeholder groups are adequately represented and broad ownership is facilitated [17,35,70,71].

KT entities based on Archetype B, organized around a logic of using designated knowledge brokers working within loosely autonomous research streams, achieve ambidexterity through the development of the absorptive capacity of numerous brokers who now have a better understanding of explorative processes [72]. Though the exploratory research process is not explicitly designed to change—as in archetype A—the sustained presence of knowledge brokers from service provider domains may nonetheless influence the research process and dynamics. Informing service providers and implementation leads regarding the rigor and process of research can support its utilization through improved ownership and awareness [42,73]. Given the key role of knowledge brokers in influencing both exploration and exploitation in Archetype B, it is important to ensure that they have sufficient ability to engage with researchers as well as steer the implementation processes. Thus, the level of power and seniority of the knowledge broker as well as their ability to develop and sustain relationships is an essential consideration.

Archetype C, involving independent research and implementation streams, is unique in its organizing logic of modular research and implementation as parallel activities. Unlike other ambidextrous strategies, there is little cross over and fertilization of ideas or organizational processes between exploratory research and exploitative implementation, a strategy that the innovation literature has highlighted as particularly efficient to coordinate [62]. In the language of systems design, exploration can be pursued in one module of a modular system while exploitation is pursued in another, in which case conflicts over resources, mindsets, and organizational routines and timeframes are less problematic [62,69]. The absorptive capacity of provider organization staff in having the skills to search for, recognize, and assess relevant research to exploit knowledge is key to sustaining ambidexterity and is of central importance for implementation leaders. As such, Archetype C’s model enables ambidexterity through the dynamic capability of both increasing the efficiency of knowledge search (*e.g.*, being able to find good evidence) and supporting the assimilation of information to specific problems within the immediate service provider context [56,59].

In Archetype D, the organizing logic is collaborating through loose networks. Ambidexterity is facilitated by capitalizing on the existing relationships and building capacity concerning each other’s work context. The relatively informal mechanisms of central governance over projects can lead to considerable variation in explorative and exploitative scope, leading to a loosely structured ambidexterity approach, as well as novel ways of integrating patients into the networks. The literature on social networks and social capital suggests that strong and dense social connections are efficient at sharing fine-grained and in-depth knowledge for exploitative learning [72]. For example, the more frequently employees interact with particular parties, the more opportunities they have to recognize and access the parties’ idiosyncratic knowledge [73,74] and mediate trust relationships. The relationships between research and implementation partners promote exploitation as it may enable the implementation requirements to be incorporated into the research design. A potentially negative consequence of strong relationships suggested in the literature is the formation of cliques and silos, especially when certain groups become unconnected in the wider network [75]. Since the Archetype D is dependent on informal links it is necessary for the leaders and central management to nurture integrative relationships across the entire network to facilitate knowledge flows [75].

KT entities organized around Archetype E logic, involving centrally controlled service improvement projects, are strongly focused on exploitation processes in addressing the KT gap. These entities achieve ambidexterity through centralized and tightly coupled management structures and diffuse knowledge through monitored networks [56]. Managed routines facilitate knowledge flows in very specific knowledge domains that are highly suited to incremental innovation and pursuing well-defined solutions [76]. Thus, the innovation emphasis is on refining and deepening existing knowledge so as to expand or enrich service provision. Within the implementation science domain, this approach has been characterized as systematic tailoring of interventions [52] and has a strong focus on overcoming barriers for change and implementation [11]. Ambidexterity is shaped by exploring how to exploit any knowledge generated; the ambidextrous capability emphasizes proximate gain for more certain outcomes with significant potential to influence the practice of service providers. The capability developed around learning to implement and evaluate services—an important area of absorptive capacity development—contributes directly to service improvement.

In this study, we do not attempt to assess whether a particular archetype is ‘better’ than another in terms of delivering KT outcomes. Each type can be managed and led more or less effectively, present different challenges, and are more suited to certain contexts. KT implementers of each of the archetypes have various ways to accomplish translation; the archetype selection is likely to be influenced by factors such as partners’ organizational structure, current capabilities of the various partners, and the legacy of collaborative relationships, as well as the nature of KT required or envisioned. The archetypes A to E uncovered by our research of the nine CLAHRCs should be considered as being on a continuum of exploration and exploitation. Archetypes A, B, and C maintain relatively high levels of exploration and knowledge generation and, to varying degrees, can support research autonomy. We contend that these KT entities are likely to produce more generalizable knowledge that has potential to contribute to addressing the KT gap in the wider healthcare system. The weaker and non-redundant social connections found in Archetypes A and B are likely to provide individuals (from research and service provider contexts) with opportunities to identify and utilize novel knowledge from a variety of sources and, thus, encourage exploratory learning. Archetypes D and E center their focus on exploitation in order to facilitate and tailor implementation in local contexts, though with differing levels of central control. In these KT entities knowledge production is more contextually based and specifically targeted at generating value at the local level [11,52], with greater potential to influence the practice of service providers and in so doing effectively span the KT gap.

We suggest that understanding and clarifying the organizing logic underpinning KT collaborations is important for two reasons. First, a clear organizing vision is important to enable leaders to unite multiple stakeholders and enable effective communication of common goals. Given the multiple challenges, as evidenced in this study and echoed in the innovation literature, an overarching vision is an essential leadership task to engage and orientate people. Without adequate clarity of purpose and focus, the bundles of activity may seem chaotic and disjointed rather than integrated. Second, a clear vision enables leaders to develop and articulate a clear strategy for achieving KT goals. Each archetype presents unique challenges and requires particular attention from leaders to sustain performance. A clear strategy enables leaders and central management to focus their resources on developing appropriate synergy so as to maximize the distinct strengths of their KT archetype.

Based on our analysis of the five archetypes, we suggest a few practical implications. Leaders of archetype A and B KT entities need to ensure that the various groups remain connected and that brokering and negotiation roles and tasks are assigned to suitable individuals. Furthermore, selected staff need sufficient time outside of their traditional full-time roles to perform the knowledge brokering roles effectively. In Archetype C KT entities the leaders should ensure that service providers responsible for implementation have the requisite training to be able to find, access and assess relevant research. Those implementing Archetype D KT entities need to ensure that networking is facilitated and managed, while recognizing that network governance is seldom enabled through traditional hierarchy and control. Leaders could, *e.g.,* ensure there are opportunities for individuals to meet, engage, and exchange knowledge and use IT systems to facilitate communication within and beyond the networks. Archetype E KT entities require a strong project management team supported by a hierarchical work culture, to ensure that the KT entity maintains its focus on ensuring effective implementation. Further, the implementation and use of an effective IT system will provide support and focus on performance metrics at a service level.

## Conclusion

Our paper contributes an understanding of five different models for organizing KT. We have shown how learning and coordination practices are different when conducting explorative or exploitative activities [56,62,69] and how these differing orientations can be approached within the various archetypes. Each archetype has its own unique strengths and also presents unique challenges which directors should pay particular attention to in order to improve the performance of their own CLAHRC. Additionally, we have provided support for previous research on the CLAHRCs, in particular the central role of knowledge brokering arrangements in managing and negotiating the research-practice boundary [18,21] and the relevance of social networks in influencing research as well as the stakeholders involved [77].

We suggest that our work on developing KT archetypes opens a number of productive research areas for the future. First, whie this research examined KT entities at one point in time, it is important to understand how KT models change and adapt over time. As some challenges, such as improved levels of absorptive capacity, may be overcome, new challenges may arise. Additionally new models for organizing may arise as leaders reflect on and evolve their organizing structure and culture. Second, further work is needed to better understand which brokering roles are more effective and what the implications of different forms of governance and accountability for improved KT are. In addition, future work should develop ways of evaluating and assessing the effectiveness and performance of KT activity that include but go beyond ‘tick-the-box’ metrics to suggest qualitative assessment of effective knowledge brokering. Finally, an emerging area of increasing importance in KT research is to understand how the advent of new policies, such as academic health science and other clinical networks influence the evolution of KT entities and their activities, thereby shaping the innovation landscape in healthcare.

## Endnotes

^a^http://www.rand.org/ The RAND Corporation is a non-profit institution that helps improve policy and decision making through research and analysis.

^b^A number of CLAHRC directors and senior team members subsequently drew on these models to reflect on revising their organizing structure in the CLAHRC 2 reapplication bid, thereby further highlighting the practical usefulness of the models.

^c^This might include bringing engineering, architecture or sociology departments into the research process, or including a police member in the stakeholder team.

^d^An example of patient involvement might include patients or their representatives examining their own role in the research process, and providing feedback on engagement processes to the ‘research team.’

^e^Which may be run in a similar way to a program grant, yet have more outside influence from brokers.

^f^For example, a broker who might have a role as manager of a stroke service would have a different ability to influence and broker the research process than a nurse or doctor involved in the stroke service.

^g^For example, if systematic reviews or reputable guidelines exist for diabetes’ community pathways, these can form the basis of the implementation focus, as they turn their attention to getting the guidelines used.

^h^For example, patients or public who have been involved with research teams, or who have strong ties to provider organizations through advocacy groups can become centrally involved in project groups.

^i^For example, a ward may seek to improve their discharge compliance having seen successful examples of this elsewhere.

## Abbreviations

CLAHRC: Collaborations for leadership in applied health research and care; DoH: Department of health; NIHR: National institute for health research; NHS: National health service; KT: Knowledge translation.

## Competing interests

The authors declare they have no competing interests.

## Authors’ contributions

EO, MB, and GR collected and analyzed the data. EO, MB and KP drafted the manuscript. All authors contributed to the development of the models. All authors read and approved the final manuscript.
